# Stretchable Loudspeaker using Liquid Metal Microchannel

**DOI:** 10.1038/srep11695

**Published:** 2015-07-16

**Authors:** Sang Woo Jin, Jeongwon Park, Soo Yeong Hong, Heun Park, Yu Ra Jeong, Junhong Park, Sang-Soo Lee, Jeong Sook Ha

**Affiliations:** 1 KU-KIST Graduate School of Converging Science and Technology, Korea University, Seoul, 136-701, Korea; 2 Department of Mechanical Convergence Engineering, Hanyang University, Seoul, 133-791, Korea; 3 Department of Chemical and Biological Engineering, Korea University, Seoul, 136-701, Korea; 4 Photo-Electronic Hybrids Research Center, Korea Institute of Science and Technology, Seoul, 136-791, Korea

## Abstract

Considering the various applications of wearable and bio-implantable devices, it is desirable to realize stretchable acoustic devices for body-attached applications such as sensing biological signals, hearing aids, and notification of information via sound. In this study, we demonstrate the facile fabrication of a Stretchable Acoustic Device (SAD) using liquid metal coil of Galinstan where the SAD is operated by the electromagnetic interaction between the liquid metal coil and a Neodymium (Nd) magnet. To fabricate a liquid metal coil, Galinstan was injected into a micro-patterned elastomer channel. This fabricated SAD was operated simultaneously as a loudspeaker and a microphone. Measurements of the frequency response confirmed that the SAD was mechanically stable under both 50% uniaxial and 30% biaxial strains. Furthermore, 2000 repetitive applications of a 50% uniaxial strain did not induce any noticeable degradation of the sound pressure. Both voice and the beeping sound of an alarm clock were successfully recorded and played back through our SAD while it was attached to the wrist under repeated deformation. These results demonstrate the high potential of the fabricated SAD using Galinstan voice coil in various research fields including stretchable, wearable, and bio-implantable acoustic devices.

In recent years, stretchable electronics for wearable and body-implantable devices have been actively studied^1^,^2^,^3^,^4^. Many types of stretchable and wearable sensors, actuators, energy storage and energy harvesting devices have been proposed^5^,^6^,^7^,^8^,^9^, since they have a huge potential in the applications of the human-friendly technology such as electronic artificial skin^5^,^6^,^10^, multifunctional systems for health diagnosis and treatment^11^,^12^,^13^ and wearable vital motion energy harvesting device^14^,^15^. In order to operate such a device successfully under the mechanical strain exerted from the outside, deformable and conformal properties are essential, since most parts of the human body such as the skin, internal organs, and the lining membrane are composed of nonplanar, soft, and deformable surfaces^16^. To achieve the goal of obtaining deformable and conformal devices, many strain-relieving approaches adopted novel design and materials where various stretchable conductors such as serpentine-shaped or pre-wrinkled interconnections^17^,^18^, liquid metals^3^,^19^,^20^,^21^,^22^, polymer-conductor (carbon nanotubes, Ag nanowires) composites^23^,^24^, and ionic conductors were used^25^.

Since the late 1800 s, acoustic devices such as the loudspeaker and microphone have been designed and developed to apply the mechanisms of dynamic^26^,^27^, electrostatic^28^,^29^, and piezoelectric acoustics^30^,^31^. Electrostatic acoustic devices generate sound waves via a vibrating charged stator by an externally applied electric field. Piezoelectric devices are operated by using the inverse piezoelectric effect. Moreover, the most widely used dynamic acoustic drivers utilize a moving metal wire coil, i.e., voice coil, which was first devised by Siemens in 1874^26^.

Considering the importance of acoustic applications such as sensing biological signals, hearing aids, and notification of information via sound, it is also essential to realize stretchable acoustic devices for wearable and body-attached applications. The recent development of fabrication technology and materials science has enabled the investigation of next-generation acoustic devices such as graphene-based flexible and transparent sound sources^29^,^30^, thermoacoustic loudspeakers^32^,^33^,^34^,^35^, and ion-gel-based transparent loudspeakers^25^. Despite such progress, however, there still exists a barrier that slows down the technological extension from flexible to stretchable acoustic devices. In particular, suitable materials that provide mechanical stability in the fabricated devices upon the drastic deformation of stretching should be newly proposed.

Galinstan, an eutectic alloy liquid metal consisting of gallium (68.5%), indium (21.5%), and tin (10%)^36^, has excellent electrical conductivity (3.46 × 10^6^ S m^−1^ at 20 °C), a low melting point (−19 °C), low vapor pressure, and low toxicity when compared with mercury^36^,^37^. Such properties enable the wide application of this Galinstan in stretchable, wearable electronics as a stretchable interconnection between active devices^3^,^20^,^38^, as an active part of the device such as pressure^39^ and strain^40^ sensors, stretchable inductors^21^, RF antennas^41^,^42^, and as an actuator for inducing chaotic advection^43^.

In this study, a simple fabrication of a Stretchable Acoustic Device (SAD) was demonstrated by replacing the conventional rigid metal coil with a deformable liquid metal coil of Galinstan in a flexible polymer microchannel where the fabricated stretchable loudspeaker is driven by the dynamic interaction between the liquid metal coil and a permanent Neodymium (Nd) magnet. The fabricated SAD exhibited very stable acoustic performance in the audible frequency range from 20 Hz to 20 kHz^44^,^45^, upon repeated cycles of mechanical deformation of uniaxial stretching up-to 50% and biaxial stretching up-to 30%. These results and the attached supplementary videos intuitively show the high applicability of the Galinstan-based SAD to various stretchable acoustic devices.

## Results

### Fabrication of stretchable acoustic device

**Figure 1:**
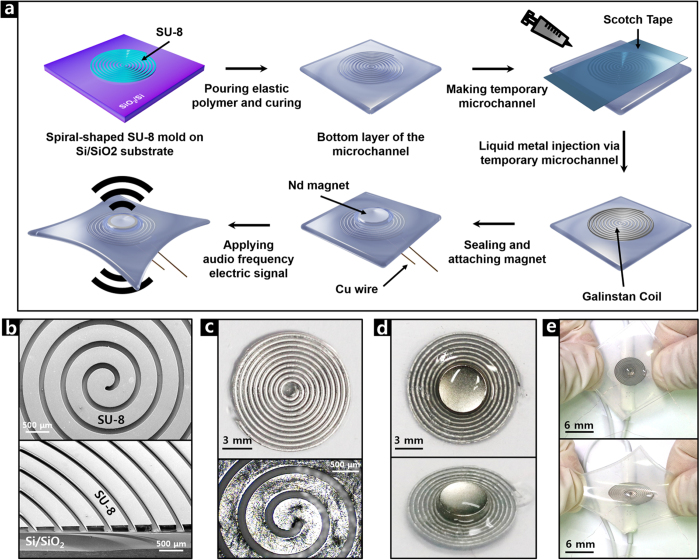
Fabrication of a stretchable acoustic device. (**a**) Schematic illustration. (**b**) SEM images of SU-8 mold. Optical images of (**c**) Galinstan microchannel, (**d**) fabricated SAD and (**e**) hand-stretched SAD.

Figure 1 shows a fabrication process of our SAD and corresponding optical images. Galinstan was injected into a spiral-shaped elastic polymer microchannel, which had been formed by pouring mixtures of polydimethylsiloxane (PDMS) and Ecoflex (MEP) over a spiral-patterned SU-8 microchannel mold produced by the photolithography process. After connecting the Cu wires with the Galinstan channel, a thin Ecoflex film was coated for sealing the channel. Then a permanent magnet of Nd was attached to the center of the embedded Galinstan voice coil in the MEP film. For a better understanding of the fabrication process, a two-dimensional representation and corresponding optical microscopic images are shown in Supplementary Fig. S1 and Fig. S2, respectively. Further details are explained in the Methods section.

### Electrical properties of liquid metal microchannel under mechanical deformation

To investigate the mechanical and electrical stability of the fabricated liquid metal coil, current–voltage (I–V) curves across the spiral-patterned Galinstan coil were measured according to the applied strain in both the uniaxial and biaxial directions. Both the uniaxial and biaxial strains are defined as shown in Supplementary Fig. S3. The resistance of the fabricated liquid metal coil is approximately 2 Ω.

**Figure 2:**
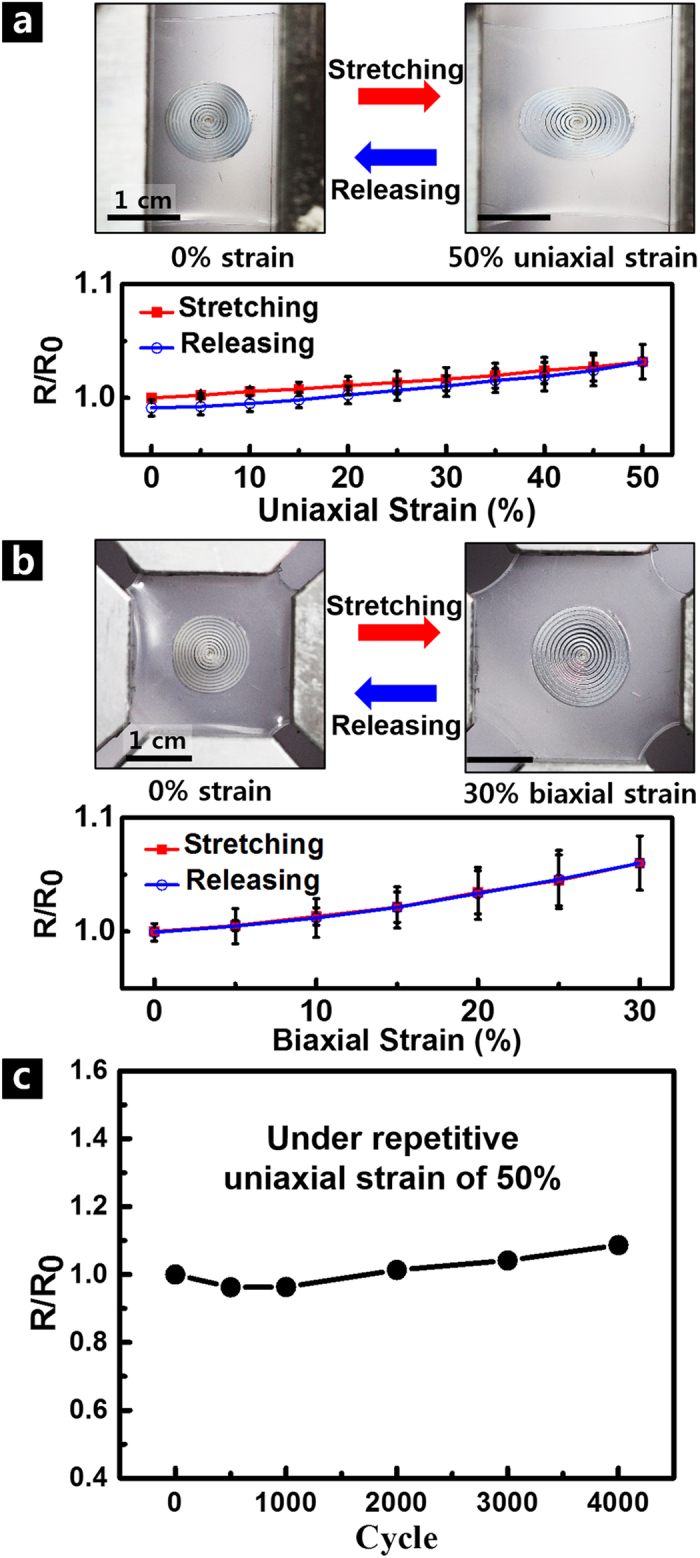
Electrical properties of the liquid metal microchannel under mechanical deformation. (**a**) (Top) optical images under uniaxial stretching and (bottom) normalized resistance (*R*/*R*_*0*_) vs. applied strain. (**b**) (Top) optical images under biaxial stretching and (bottom) normalized resistance (*R*/*R*_*0*_) vs. applied strain. Here, *R*_*0*_ and *R* are the resistance of the liquid metal coil before and after the application of strain, respectively. Red and blue symbols represent data obtained while the device is stretched and released, respectively. (**c**) Change of normalized resistance (*R*/*R*_*0*_) with repetition cycles of 50% uniaxial stretching.

Figure 2(a,b) show that the resistance increases gradually with the extent of stretching, reaching an increase of 6% with both uniaxial stretching of 50% and biaxial stretching of 30%, without hysteresis. Figure 2(c) shows the change in normalized resistance (*R*/*R*_*0*_) with respect to the repetition cycle of uniaxial strain of 50%: There is almost no change in *R*/*R*_*0*_ up to 2000 cycles, but a slight increase thereafter, reaching a ~10% increase upon 4000 cycles of stretching, was observed. Such an increase is mainly attributed to the increase of the length and the decrease of the cross-sectional area of the Galinstan coil^39^,^46^. In addition, any possible change in the contact resistance between the liquid metal and the copper wire due to the mechanical deformation or due to alloy formation of Galinstan with other metals^21^,^47^, might have contributed to it. The resistance might be affected by the air bubbles formed inside the microchannel, which can be solved by improving the sealing method, as described in Supplementary Fig. S2.

### Acoustic performance of SAD as a loudspeaker

**Figure 3:**
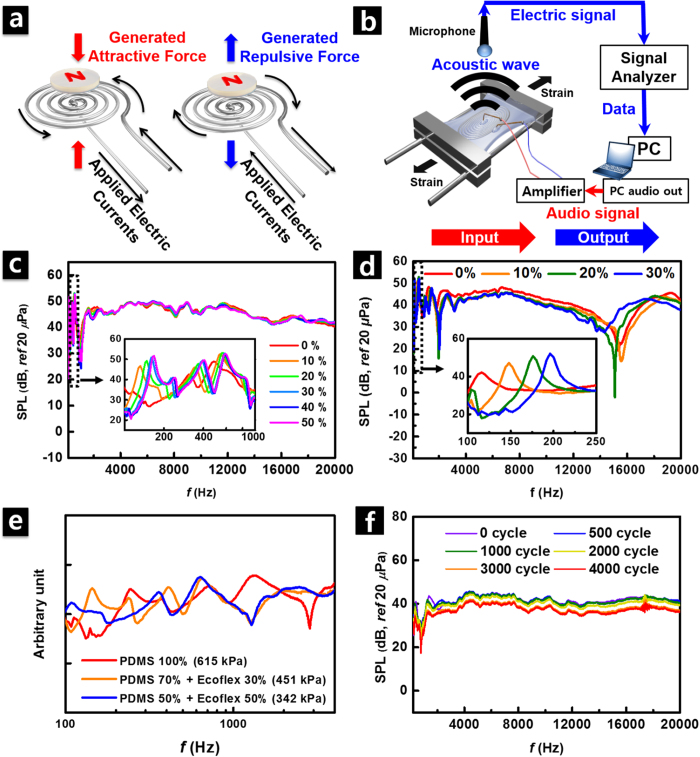
Acoustic performance of SAD as a loudspeaker. (**a**) Operating principle of SAD as a loudspeaker. (**b**) Schematic illustration of measuring frequency response of fabricated SAD as a loudspeaker. (**c**) Change of sound pressure level (SPL) vs. frequency with variation of uniaxial strain from 0 to 50%. Inset is zoomed data in low-frequency region of 100–1000 Hz. (**d**) Frequency response curves taken under biaxial strain in wide range of frequencies (100 Hz–20 kHz). Inset is zoomed data in low-frequency region of 100–250 Hz. (**e**) Spectral characteristics taken from SAD with different diaphragm materials. Red, orange, and blue are for PDMS, composite of PDMS and Ecoflex with a mixing ratio of 7:3, and composite of PDMS and Ecoflex with a mixing ratio of 1:1, respectively. Their corresponding Young’s modulus are 615, 451, and 342 kPa, respectively. (**f**) Change of SPL vs. frequency with repeated number of cycles under 50% uniaxial stretching.

Figure 3(a) shows a schematic illustration of the operating principle of the fabricated SAD as a loudspeaker. According to the driving principle of the dynamic electroacoustic transducer, the Lorentz force (F) is generated in the current-carrying liquid metal wire following the Equation (1)


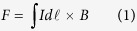


where *ℓ* is a vector whose magnitude is the length of the wire (*dℓ* is an infinitesimal segment of wire.), *B* is the intensity of the magnetic field, and *I* is the steady current flowing along the wire. The direction of the current changes depending on the audio frequency electric signal applied from the outside. This change in the direction of the current causes an alternate change in the direction of the vertical Lorentz force. Thus, a sound wave is generated from such repetitive vibration^27^.

Additionally, to show that the sound waves are formed by the electromagnetic interaction of a magnet and the Galinstan coil, a simple intuitive demonstration was performed as shown in Supplementary Video S1. While the piano melody played by one of the authors was applied to the Galinstan coil and the position of the Nd magnet was varied, the sound generation was observed. As expected, sound wave was generated more effectively when the magnet was positioned closer to the coil. By doing this, it can be easily checked that the fabricated device is operated in a dynamic way. In addition, the generation of sound waves by the Galinstan coil with attached magnet can be also confirmed.

Figure 3(b) shows experimental setups for measuring the spectral characteristics of sound radiation from our SAD as a loudspeaker. The input sine sweep signal, with a frequency ranging between 20 Hz and 20 kHz, was applied to our transducer through the signal generator, and the generated sound pressure was measured by using a microphone that was positioned 1 cm away from the center of the vibrating surface. In order to avoid possible overwork of the sound analyzer and fabricated transducer, the amplitude of the input voltage signal was amplified to ~1 V (0.3 A)—just enough to recognize the spectral characteristics. The sound pressure level (SPL) measured from the fabricated device showed relatively flat response characteristic over a wide frequency range (see Fig. 3(c)), which is preferred for human audible acoustic actuators since the ideal loudspeaker must radiate constant SPL in the audible frequency range (20 Hz–20 kHz)^29^. Thus, our SAD has advantages over thermoacoustic transducer since the latter does not produce sufficient sound pressure due to the reduction in power efficiency from ~10^−6^ (at 20 kHz) to ~10^−8^ (at 3 kHz)^29^,^32^,^48^. This is caused by heat dissipation in the audible frequency band^32^.

Under uniaxial strain of up to 50% (see Fig. 3(c)) and a biaxial strain of up to 30% (see Fig. 3(d)), the fabricated SAD successfully maintained its performance without any noticeable deviation in the frequency range from 1 to 20 kHz. Due to the structural configuration of the device, the polymer substrate of the elastic microchannel exhibited the mechanical characteristics of a diaphragm. The modal characteristics of the fabricated SAD were significantly affected by the applied axial load at the edges, and the applied axial load increases the natural frequencies. The measured SPL in the relatively low frequency range from 100 to 1000 Hz (insets) showed the influence from the modal response with increase of the resonance frequencies^49^. Furthermore, the acoustic performance of the device was also strongly influenced by the elastic modulus of the substrate material as shown in Fig. 3(e).

Red, orange, and blue are for PDMS, composite of PDMS and Ecoflex with a mixing ratio of 7:3, and composite of PDMS and Ecoflex with a mixing ratio of 1:1, respectively. The weight mixing ratio of MEP film can be changed depending on the desired extent of deformation due to the difference in the Young’s modulus between Ecoflex (69 kPa^20^) and PDMS (615 kPa^3^). Here, the modulus for the composite material was calculated by using the relation of G_mix_ = G_PDMS_f_PDMS_ + G_Ecoflex_f_Ecoflex_, where G is the initial modulus and f is the volume fraction^50^. Such calculated Young’s modulus for corresponding materials are 615, 451, and 342 kPa, respectively. In general, vibration and acoustic emission characteristics of the structure are affected by the bending stiffness (*D*) associated with the elastic modulus (*E*) of the constituent material as shown in Equation (2)


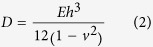


where *E* is the elastic modulus of the constituent material, *h* is the thickness and *v* is the Poisson’s ratio^51^. For a rectangular-shaped SAD fabricated in our research, the frequency characteristic was changed in accordance with the change of the substrate material as shown in Fig. 3(e). The resonance frequency increased with the increasing Young’s modulus of the substrate material. As for the axial load applied to the device, the acoustic performance of the SAD was closely associated with the mechanical properties of the substrate, determining the radiating frequency and amplitudes. Thus, elastic modulus of the substrate material must be deliberately considered in order to improve the acoustic performance. Furthermore, the total thickness of the elastomer film also affects the acoustic performance of the device and changes the spectral characteristics. Certainly, the SAD stiffness is influenced by the properties of constituting materials including the thickness of the bottom, microfluidic and top layers. Consequently, the acoustic emission characteristics are influenced by the effective stiffness. The detailed relationship between the acoustic characteristics of the materials and individual thickness of constituting materials can be predicted and optimized by calculating the equivalent stiffness of the multilayer according to the previously reported work^52^. However, in this work, we focused on understanding the influence of the substrate material properties while keeping the thickness of constituting layers constant (Bottom layer: 700 μm, Sealing layer: 300 μm) rather than investigating the influence of the layer thickness. Reducing the device thickness is one of the important issues in the application of the wearable and bio-implantable electronics since epidermal devices require ultrathin thickness. If the thickness of the liquid metal wire became thinner, the resistance of the coil would be increased. As a result, it is expected that the electric current flowing through the coil would be reduced under the same input voltage signal applied. Reduction of current (*I*) consequently causes a reduction in the Lorentz force (*F*), which leads to a reduction in acoustic performance in broad frequency range as shown in the Equation (1). However, such problem can be solved via increasing the input voltage signal to compensate the reduced current. Furthermore, decrease in the thickness of the liquid metal coil facilitates the reduction of the total thickness of the substrate polymer, leading to enhance the acoustic performance owing to the increase in the average vibration velocity in the low frequency range^53^. In future research, it is expected that desired modal properties of the diaphragm can be acquired by varying the material and the thickness of the elastic polymer microchannel. In addition, further studies on the coupling effect of the liquid metal coil and the elastomer film would be helpful to realize higher-performance thinner devices by optimizing the structure of the device components.

The boundary conditions depending on the fixing method of the SAD to the stretching stage seemed to affect the spectral characteristics of the SAD, as shown in Supplementary Fig. S4^54^.

The moderately reliable performance of SAD, even during 4000 cycles of repeated stretching of 50% uniaxial strain, was confirmed in Fig. 3(f): Up to 2000 cycles, the deviation in the radiated SPL was minimal as less than 2–3 dB in the entire frequency range. However, after 2000 cycles, due to the increased resistance of the Galinstan coil as shown in Fig. 2(c), the radiated pressure showed a slight reduction as the repetition cycles increased. However, since the spectral response is determined by inherent characteristics of the entire system including a magnet, polymer substrate, and Galinstan coil, the sound radiation was not affected significantly by the repetitive cycles of stretching.

**Figure 4:**
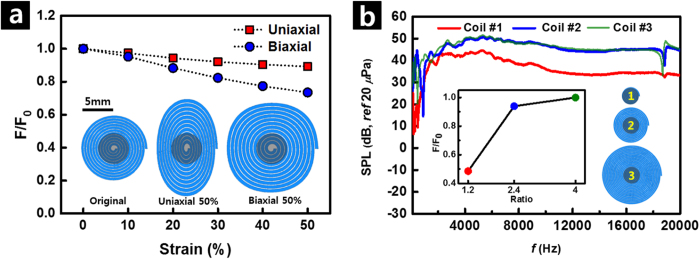
Acoustic performance of SAD evaluated via FEM analysis. (**a**) Change of normalized Lorentz force (*F*/*F*_*0*_) with increase in uniaxial (red) and biaxial (blue) stretching. *F*_*0*_ and *F* are the Lorentz force before and after application of strain, respectively. (**b**) Change of SPL vs. frequency with change in size of liquid metal coil. Inset is the change of *F*/*F*_*0*_ with change in ratio of coil diameter to diameter of magnet. Here, red, blue, and green correspond to the inset picture of 1, 2, and 3, respectively.

The Lorentz force and the generated sound pressure are related by the Equation (3).


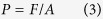


where *P* is the pressure, *F* is the vertical component of the Lorentz force, and *A* is the area of the surface. Assuming that *A* is a constant, *P* is influenced only by *F*. In order to estimate the effect of coil deformation on the device performance, the Lorentz forces generated upon an application of 50% uniaxial and 30% biaxial strain were calculated by finite element method (FEM) analysis. The decrease of the Lorentz force with the applied strain is expected, because the distance between the liquid-metal conductor and the center of the coil where the Nd magnet is positioned increases^27^. Figure 4(a) shows the change in the normalized Lorentz force (*F*/*F*_*0*_), where *F*_*0*_ and *F* are Lorentz forces before and after application of the strain, upon increasing the applied strain in uniaxial (red) and biaxial (blue) directions, respectively. *F*/*F*_*0*_ decreased by 10.7% and 17.7% under 50% uniaxial and 30% biaxial stretching, respectively. Since the rigidity of the Nd magnet was not considered for the estimation, those changes are the upper limit of the variation in the Lorentz force induced by the applied strain. Therefore, in a real situation, a much smaller strain can be applied to the central liquid-metal-injected microchannel^3^, which is close to the rigid magnet, forming a larger Lorentz force than that in the outer part. Even the upper-limit changes in the Lorentz force correspond simply to a difference in SPL of 1–2 dB^54^. Since the minimum noticeable change in SPL by the human ear is ~3 dB^55^, the effect of the change in the Lorentz force due to stretching deformation on the actual acoustic devices seems to be negligible. These results are consistent with the actual device performance shown in Fig. 3(c–d), where no noticeable change with both uniaxial and biaxial stretching was observed.

Next, the influence of the number of turns of Galinstan coil on the frequency response was investigated by using three different dimensions of the coil, as summarized in Table 1. Figure 4(b) shows the higher SPL when the coil diameter was much larger than the magnet diameter (coils #2 and #3). It is attributed to the increased Lorentz force (F) resulting from an increased number of turns of Galinstan coil causing an increase in 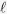-value of the Equation (1). However, as the ratio of the coil diameter to the magnet diameter becomes larger, the SPL is saturated, attributed to the smaller contribution of the Lorenz force induced by the Galinstan coil that is distant from the center-positioned Nd magnet. The similar behavior of the calculated Lorentz force to the measured SPL was obtained through FEM analysis, as shown in the inset of Fig. 4(b).

### Acoustic performance of SAD as a microphone

**Figure 5:**
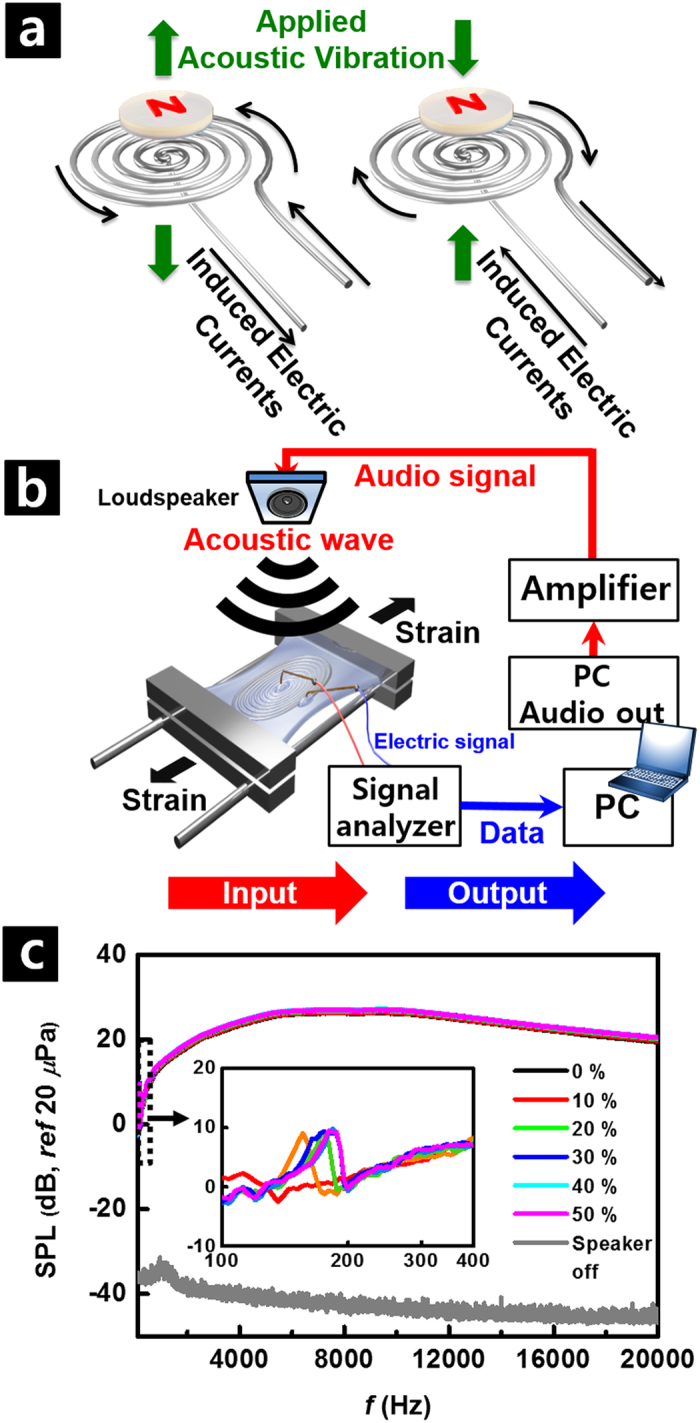
Acoustic performance of SAD as a microphone. (**a**) Operating principle of SAD as a microphone. (**b**) Schematic illustration of measuring sensing capability of fabricated SAD as a microphone. (**c**) Change of detected SPL vs. frequency with variation of uniaxial strain from 0 to 50%. Inset is zoomed data in low-frequency region of 100–400 Hz.

The electromagnetic interaction between the magnet and the liquid metal coil enables the application of the SAD as a microphone as well as a loudspeaker and its detailed operational principles are shown in Fig. 5(a). The induced electromotive force with audio frequency is generated by Faraday’s law of electromagnetic induction when external vibration is applied to the device^56^. By recording the induced current of the liquid metal coil, the functioning of our SAD as a microphone could be measured as schematically illustrated in Fig. 5(b). The sine-sweep voltage input in the audio frequency band was supplied to a commercial loudspeaker through the amplifier. The sound pressure generated by the commercial loudspeaker was recorded again using a fabricated transducer, which was positioned 1 cm away from the loudspeaker. The frequency of the induced current generated by the applied external sound wave is equal to that of the applied acoustic signal. Operation of our SAD as a microphone could be clearly seen by comparing the signal when the loudspeaker was off (see Fig. 5(c)). In addition, the measured spectrums did not show any noticeable deviation with an applied uniaxial strain of up to 50% in the high-frequency range. In particular, very flat response was observed in the frequency range of 1–20 kHz, suggesting linear response of the sensor. However, the shift of the resonance peaks is also observed in the low-frequency range of 100–400 Hz due to the modal characteristics of the polymer substrate (inset of Fig. 5(c)).

Comparison of our SAD with commercial acoustic devices is shown in Supplementary Fig. S5. The lower performance of the fabricated SAD can be improved by optimizing the design of the device by using a stronger magnet, reducing the thickness of the polymeric substrate, adopting a suspension structure^57^, and by applying a higher number of turns per unit area of the voice coil, while maintaining the two-dimensional film structure for its application as a wearable and bio-implantable acoustic device^27^.

### SAD as a stretchable body-attached acoustic device: Recording of sound

**Figure 6:**
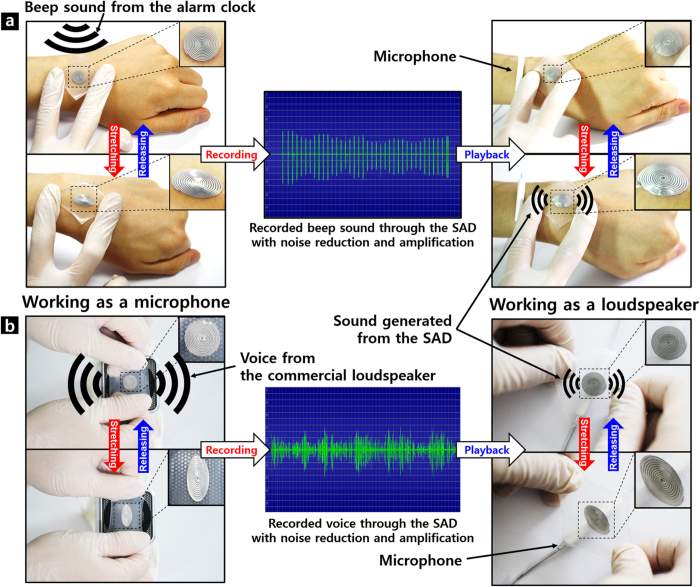
Demonstration of SAD as a body-attached device. (**a**) Recording and playback of beep sound from alarm clock via the same fabricated SAD. (**b**) Recording and playback of voice from loudspeaker via the same fabricated SAD.

**Figure 7:**
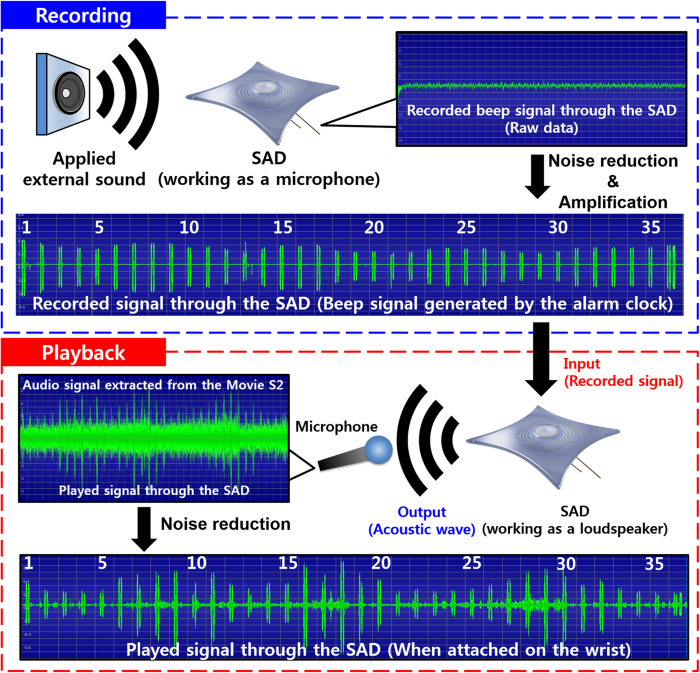
Experimental details of Supplementary Video S2 and S3 with comparison of recorded beeper signal with replayed signal.

Figure 6 schematically demonstrates the application of our SAD as a stretchable body-attached acoustic device. After attaching the device to the wrist, the beeper sound of an alarm clock [top left, Fig. 6(a)] or one of the authors’ voice reading the abstract of this paper from a commercial loudspeaker [bottom left, Fig. 6(b)] was recorded through our SAD during cycles of its stretching and releasing. The SAD connected to the microphone jack of the desktop computer worked as a microphone. Then, the noise was removed and the amplitude of the recorded sound was amplified by using the digital audio editor (Goldwave) as shown in the Fig. 7. The high-pitched electronic sound was generated at this step owing to the technical limits of noise reduction by the computer software we used.

### SAD as a stretchable body-attached acoustic device: Play-back of recorded sound

The sound recorded by the SAD was replayed by the same fabricated device (right top and bottom of Fig. 6). Here, the SAD connected to a speaker terminal of the computer operated as a sound source. The input signal was amplified using a commercial loudspeaker to obtain a sufficient acoustic output in the playing-back step. The output acoustic wave from the SAD was recorded again by using a commercial microphone. Supplementary Video S2 and S3 clearly show that our SAD works successfully while it is stretched on the wrist or stretched by hand. The electronic sound generated during the noise removal and amplification process was heard loudly when the recorded signal was played back. However, when the original voice signal was applied to the device, much louder and clearer sound was generated. It was also confirmed in the Supplementary Video S3. The replayed sound is compared with the recorded one with detailed experimental procedures in Fig. 7. Comparison of the input and output signals clearly showed that the period and the number of beep corresponded exactly. However, the amplitude of the output pulse signal was not uniform mainly because the distance between the SAD and the microphone was irregularly varied according to the movement of the wrist. Therefore, when the SAD was stretched by hand while maintaining a rather constant distance from the microphone, more uniform amplitude of the output signal was observed as shown in Supplementary Fig. S6 and Supplementary Video S2. During the demonstration, a larger magnet with a diameter of 7 mm and thickness of 2 mm was applied in this stage to obtain a stronger signal as a microphone, and to generate a stronger sound pressure as a loudspeaker.

In summary, we successfully demonstrated the facile fabrication of a stretchable dynamic acoustic device using a planar voice coil of liquid metal, Galinstan. As a dynamic acoustic transducer, the SAD worked simultaneously as a loudspeaker and a microphone. The fabricated SAD showed stable electric and acoustic performance under repeated application of uniaxial and biaxial strains. This was consistent with the theoretically estimated variation of the Lorenz force upon mechanical deformation. Successful demonstration of recording and playback of various types of sound under mechanical deformation shows the high applicability of our SAD as a body-attached acoustic device. It is also expected that our invention would provide a promising solution to the research fields for the stretchable, wearable and bio-implantable acoustic electronics.

## Methods

### Fabrication of flat spiral-patterned SU-8 mold

SU-8 is a highly viscous epoxy material used as negative photoresist. SU-8 is useful for making high-aspect ratio structures. First, 150-μm-thick SU-8 film was formed on a SiO_2_/Si substrate via two-step spin-coating: 500 rpm at 10 s, and then 800 rpm at 30 s. The spin-coated SU-8 film was heated on a hot plate at 95 °C for 1 h, and then kept at room temperature to cool for approximately 1–3 min. After that, the film was exposed to UV for 27 s while it was masked with a spiral-patterned photomask, forming a strong acid. The acid-catalyzed, thermally driven cross-linking of epoxy could be performed via a post-exposure bake step of 65 °C for 1 min and 95 °C for 5 min, with subsequent cooling to room temperature for 1–3 min. During the post-exposure bake step, the spiral pattern could be observed in the SU-8 film. By stirring the spiral-patterned SU-8 film in the developer, 1-methoxy-2-propanol acetate solution, for 30 min and rinsing with IPA for 10 s, the spiral-patterned microchannel mold could be obtained. The height, width, and spacing of the microchannels are 150, 350, and 100 μm, respectively.

### Fabrication of deformable polymer microchannel

A mixture of Ecoflex (0030, Smooth-On) and PDMS (Sylgard 184, Dow Corning) with a weight mixing ratio of 3:7 was poured onto the SU-8 (3050, Microchem) microchannel mold, which was kept in vacuum for 10 min to remove air bubbles. After that, it was cured in a dry oven at 65 °C for 30 min.

### Injection of liquid metal into microchannels

A piece of Scotch tape was attached over the bottom layer of the microchannel to form a temporary microchannel. Then, Galinstan was injected with a micro syringe into the temporary microchannel. Here, excessive pressure should be avoided not to cause damage to the microchannel. To prevent the channel from being damaged, a glass plate was placed on top of the microchannel except for the air outlet in the center. Through the injection and removal of the Scotch tape, a liquid metal coil contained within the bottom layer of the microchannel could be formed. The surplus Galinstan overflowing in the area between neighboring channels should be removed by rinsing with ethanol.

### Electrical wiring

Copper wires with a diameter of 100 μm were installed at both ends of the Galinstan-filled microchannel for electrical signaling.

### Sealing and Attachment of magnet

Thin Ecoflex film was coated on top of the bottom layer of the microchannel filled with Galinstan for complete sealing. Then it was cured at room temperature for 1 h. Additional sealing was performed by dropping Ecoflex at both ends of the coil and subsequently curing at room temperature for 15 min. A Neodymium magnet was attached to the center of the spiral coil using a drop of Ecoflex cured at room temperature for 30 min to provide a bonding layer between the magnet and the sealing layer. At this stage, the high temperature treatment above 65 °C was avoided since Gallium-based eutectic alloy was reported to be highly corrosive at high temperatures^58^,^59^. Therefore, we used Ecoflex as a sealing layer which can be cured rapidly at room temperature while PDMS requires much higher temperature for curing. Furthermore, we tried to avoid the phenomena of the thermal expansion and contraction of the liquid metal^60^, which may induce non-uniform structures of the microchannel and incomplete filling of the liquid metal since the shape of the micro-channel is determined by the shape of the liquid metal and the uncured polymers surrounding it.

### Measurement of electrical resistance

Electric current over the Galinstan coil was measured at every 40 mV potential interval while voltage was swept from −2 V to 2 V at both ends of the copper electrodes using a Probe Station (MS-Tech Model 8000). Resistance was calculated from the reciprocal of the slope of the measured I–V curve. The electrical performance was measured while external strain was applied by using a custom-built stretching device.

### Measurement of spectral characteristics of the fabricated SAD

Radiated sound from the SAD was recorded and evaluated using a commercial FFT analyzer (B&K Pulse LabShop), while swept-sine voltage in the auditory frequency range from 20 Hz to 20 kHz was applied to the transducer. A sound generated from the transducer was measured by using a microphone (B&K type 4189), which was located 1 cm away from the center of the vibrating surface. All of the measurements were conducted in an anechoic chamber to minimize the reflection from boundaries and to minimize the background noise. Additionally, the frequency response measurements under applied strain were conducted using a custom-built stretching device. Uniaxial strain up to 50% and biaxial strain up to 30% were applied. In addition, the change in the SPL with repetitive cycles of uniaxial stretching to 50% was measured.

## Additional Information

**How to cite this article**: Jin, S. W. *et al*. Stretchable Loudspeaker using Liquid Metal Microchannel. *Sci. Rep*. **5**, 11695; doi: 10.1038/srep11695 (2015).

## Supplementary Material

Supplementary Information

Supplementary Video S1

Supplementary Video S2

Supplementary Video S3

## Figures and Tables

**Table 1:** Specifications of coil according to number of turns and diameter.

Number	Number of turns	Diameter[cm]	Ratio of coil diameter to diameter of magnet (d=0.5)
1	5	0.6	1.2
2	11	1.2	2.4
3	18	2	4.0
