# Supplementary material for: Stretchable Loudspeaker using Liquid Metal Microchannel
Source: Sci Rep. 2015 Jul 16;5:11695. doi: 10.1038/srep11695 (PMC4504143; doi:10.1038/srep11695)
Supplement: Supplementary Information [file srep11695-s1.pdf]

Supplementary Information

Stretchable Loudspeaker using Liquid Metal Microchannel

Sang Woo Jin[1], Jeongwon Park[2], Soo Yeong Hong[3], Heun Park[3], Yu Ra Jeong[3], Junhong Park[2], Sang-Soo Lee[1,4] and Jeong Sook Ha[1,3*]

[1]KU-KIST Graduate School of Converging Science and Technology, Korea University, Seoul, 136-701, Korea

[2]Department of Mechanical Convergence Engineering, Hanyang University, Seoul, 133-791, Korea

[3]Department of Chemical and Biological Engineering, Korea University, Seoul, 136-701, Korea

[4]Photo-Electronic Hybrids Research Center, Korea Institute of Science and Technology, Seoul, 136-791, Korea.

*Corresponding author email: jeongsha@korea.ac.kr

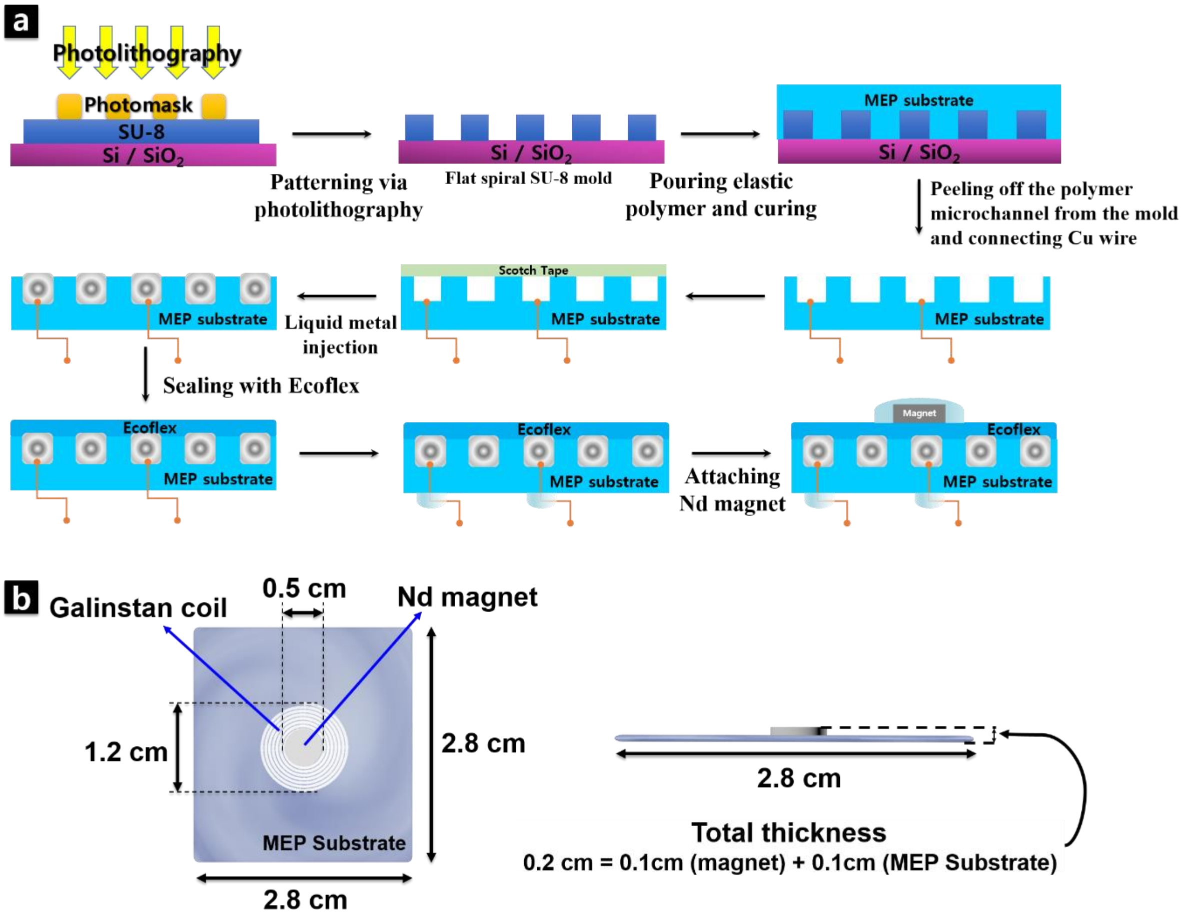

Figure S1. (a) Schematic illustration of fabrication of SAD shown in side view. (b) Corresponding dimensions of component materials.

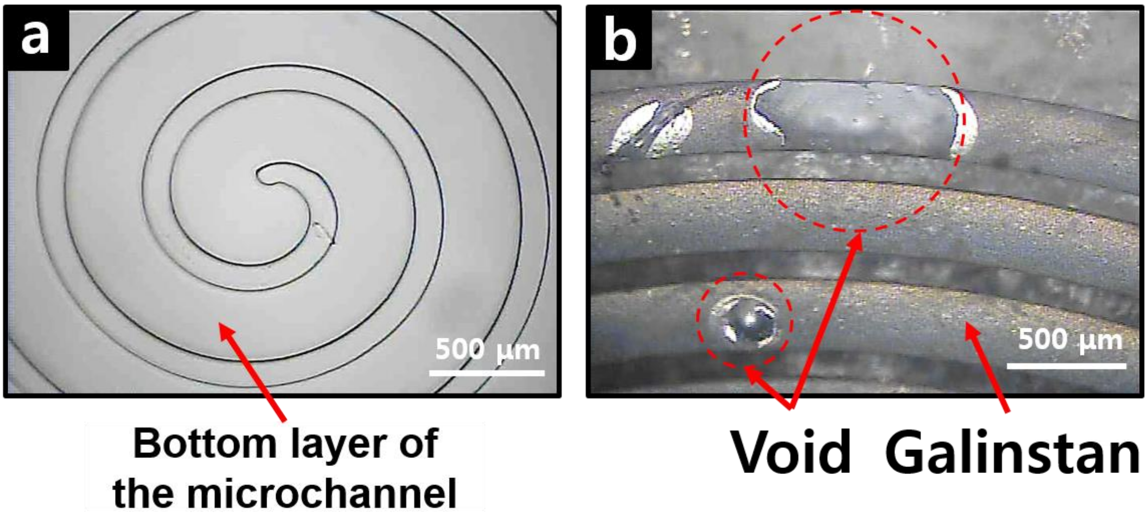

Figure S2. Optical microscope images of (a) bottom layer of the elastic polymer microchannel and (b) void formed in the microchannel.

Observed Galinstan void (**Supplementary Fig. S2 (b)**) is mainly attributed to the imperfect sealing of the microchannel, the redistribution of Galinstan upon applied external strain, and the possible air bubbles nearby the inserted Cu wire in the channel.[1] Such formed void caused the increase in the resistance. Therefore, additional process was done for better sealing of the channel: A few drops of Ecoflex were put at both ends of the Galinstan channel so that the leakage of Galinstan from the gap between the inserted Cu wires and the polymer substrate could be prohibited even when external mechanical strain was applied.

Since the spiral channel has sharp edges with a rectangular cross-section rather than a circular one, the liquid metal can be made to fill the microchannels better by applying pre-strain several times before pouring Ecoflex on top of the bottom layer of the microchannel filled with Galinstan as illustrated below.

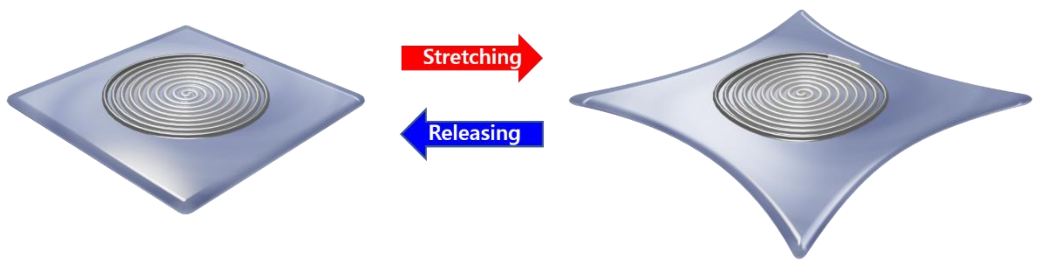

Additionally, temporary microchannel method using a scotch tape is applied to minimize the unfilled sharp edges of the microchannel by pouring the Ecoflex to the Galinstan coil as shown below.

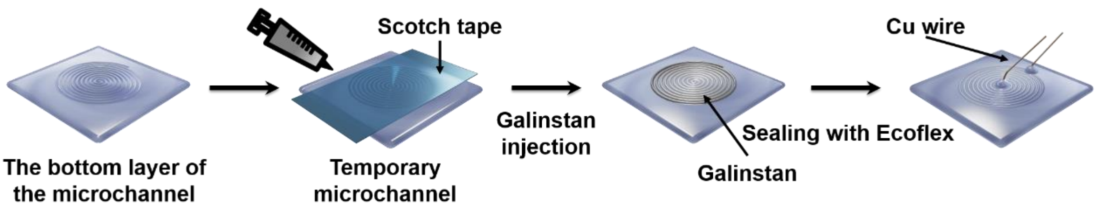

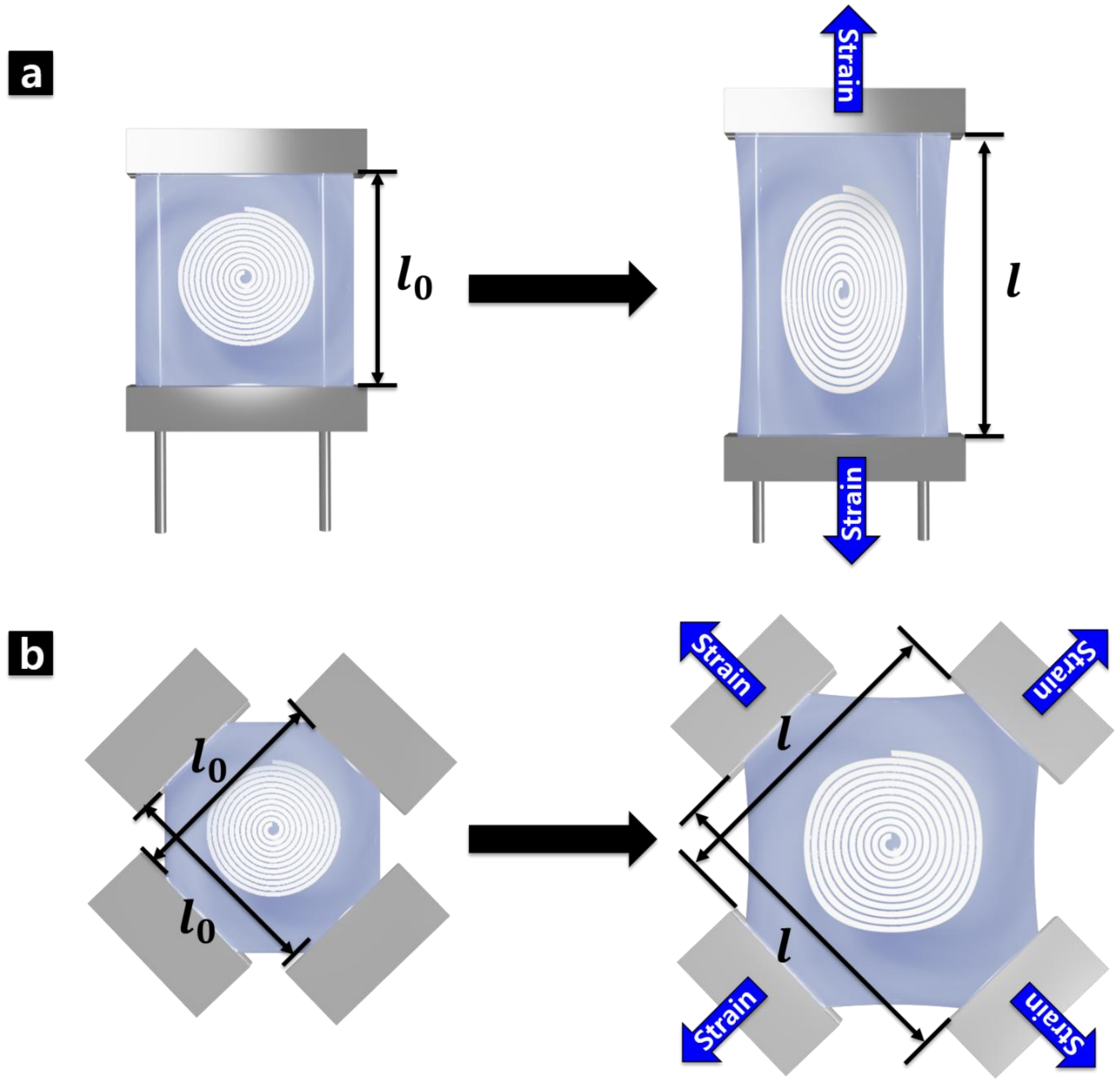

c

$$\textbf{Applied Strain}\ (\%) = \frac{l - l_0}{l_0} \times 100$$

Figure S3. (a) Definition of applied uniaxial strain on SAD.[2] (b) Definition of applied biaxial strain on SAD.[3] (c) Corresponding expression in the strain calculation.[2,3] Here, l is the length between the fixed edges after being stretched, and l_0 is the length between the fixed edges before being stretched.

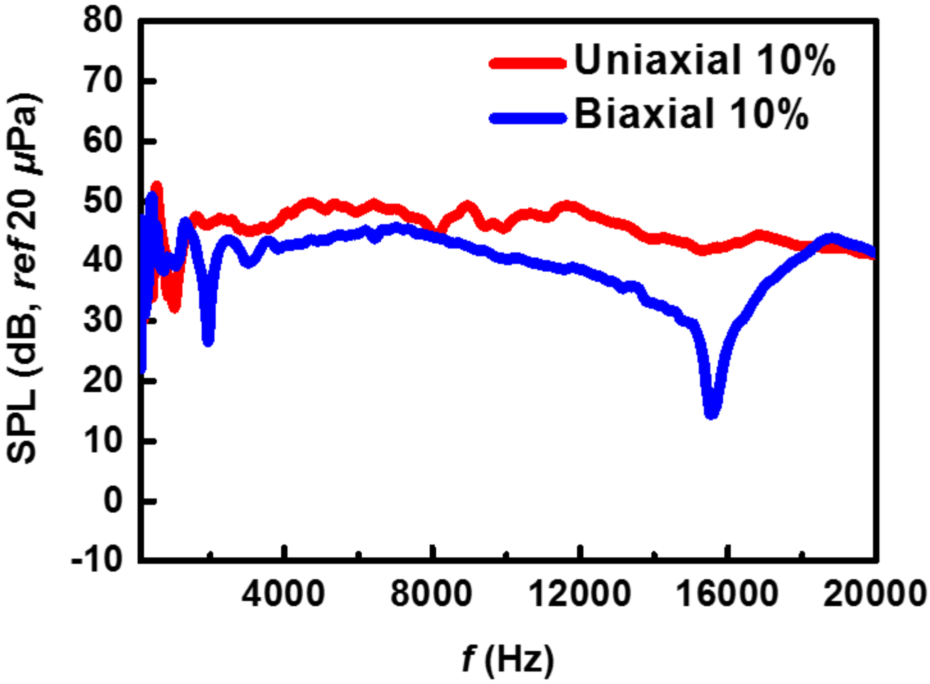

Figure S4. Comparison of SPL curves taken under 10% uniaxial (red) and biaxial (blue) strains.

Figure S4 shows that the spectral characteristic of the SAD is strongly influenced by the boundary conditions according to the method for fixing the fabricated device to the stretching stage as mentioned in the text.

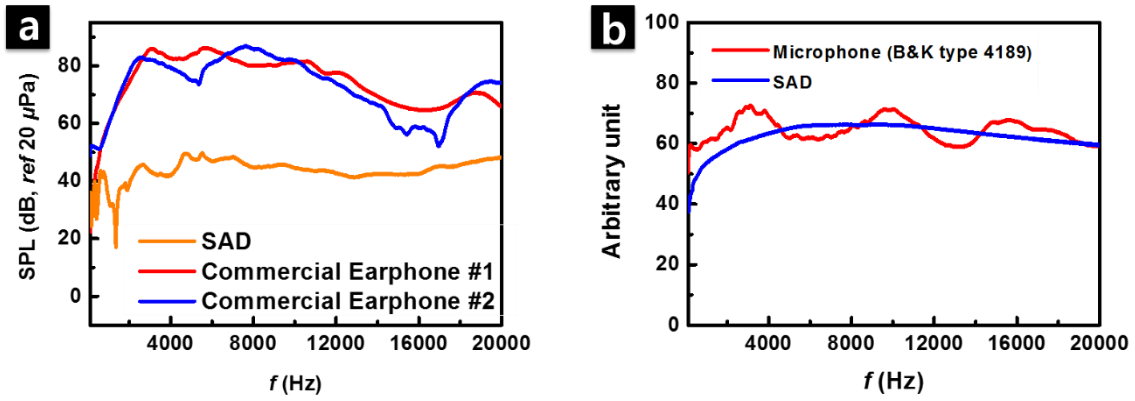

Figure S5. Comparison of acoustic performance between fabricated SAD and commercial device of (a) earphone and (b) microphone. Commercial earphone #1 and #2 are Sony XBA-C10 and Apple Earpods, Respectively.

Comparison of our SAD with a commercial earphone: A swept-sine voltage signal (mean value of 1 V) was applied to the tested device and the earphone. The generated sound was measured with the microphone 1 cm away from the center of the transducer. As commercial earphones, Sony XBA-C10 (red) and Apple Earpods (blue) were used.

The commercial earphone exhibits a higher SPL than the fabricated transducer in **Supplementary Fig. S5 (a)**. This can be attributed to several possible reasons. The commercial voice coil has a much larger number of turns than our fabricated Galinstan coil. Thus, the bigger Lorentz force (*F*) is generated due to a larger ℓ-value in the **Equation (1)**. Furthermore, the suspension structure between the voice coil and a magnet can also generate a larger pressure from commercial earphones.[7]

Comparison of our SAD with a commercial microphone: The swept-sine voltage in the audio frequency range was supplied as an input to the commercial loudspeaker. The signal was

amplified by the power amplifier (B&K type 2719). The sound generated by the commercial loudspeaker was recorded using a fabricated transducer, which was positioned 1 cm away from the loudspeaker.). Performance of the fabricated SAD was compared with that of a commercial microphone in **Supplementary Fig. S5 (b)** Due to low sensitivity of the fabricated SAD compared to the commercial microphone (45.2 mV/Pa), the sensitivity was arbitrarily set to be 1 V/Pa and the dynamic performances of the two devices were compared. The measured sound spectrum from the fabricated transducer is flatter than that of the commercial microphone, indicating the higher sensitivity of the commercial ones to better detect the frequency characteristics of the loudspeaker. The lower sensitivity of the fabricated SAD can be improved by optimizing the design of the device by reducing the thickness of the polymeric substrate or by adopting a suspension structure between the magnet and the liquid metal coil.[4,7] However, in this work, a suspension structure was not used so that the entire device would have a two-dimensional form of films in order for it to be applied to bio-implantable, wearable, and stretchable electronics.

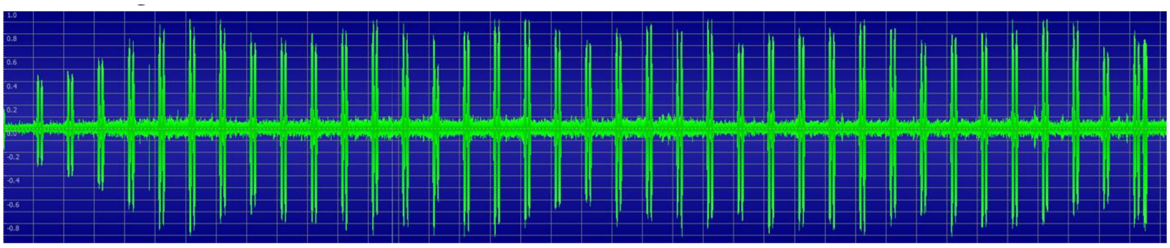

Figure S6. Recorded signal through a microphone (Beep signal replayed by the SAD when stretched by hand in Supplementary Video S2)

Supplementary Video S1

1. Simple demonstration
   - Dynamic acoustic device.
2. After attaching Nd magnet.

Supplementary Video S2

1. SAD working as a microphone (Recording beep sound)
2. SAD working as a loudspeaker (Playback of the recorded signal)
   - Stretched on the wrist.
3. SAD working as a loudspeaker (Playback of the recorded signal)
   - Stretched by hand.

Supplementary Video S3

1. SAD working as a microphone (Recording the voice from the loudspeaker).
2. SAD working as a loudspeaker (Playback of the recorded voice signal).
3. SAD working as a loudspeaker (Playback of the original voice signal).

References

[1] Lazarus, N., Meyer, C. D., Bedair, S. S., Nochetto, H. & Kierzewski, I. M. Multilayer liquid metal stretchable inductors. *Smart Mater. Struct.* **23**, 085036 (2014).

[2] Hong, S. Y. *et al.* High-Density, Stretchable, All-Solid-State Microsupercapacitor Arrays. *ACS Nano* **8**, 8844-8855 (2014).

[3] Lim, B. Y. *et al.* Biaxially Stretchable, Integrated Array of High Performance Microsupercapacitors. *ACS Nano* **8**, 11639-11650 (2014).

[4] Hwang, G. Y. *et al.* Analysis of a dynamic speaker in mobile phones by considering mechanical, electrical, and magnetic coupling effects. *J. Appl. Phys.* **91**, 6979-6981 (2002).

[5] Kenney, C. R. *Magnetic flux sensor for hearing aid application*, The University of Maine, (2005).

[6] Jung, J. H. *et al.* Lead-Free NaNbO3 Nanowires for a High Output Piezoelectric Nanogenerator. *ACS Nano* **5**, 10041-10046 (2011).

[7] D. North, inventors; Wire suspension for speakers. United States patent US 8,155,372 B2. 2012 Apr 10.
